# Mitochondria-enriched nanovesicles: A novel approach for treating radiation-induced skin injury

**DOI:** 10.1016/j.mtbio.2025.102377

**Published:** 2025-10-08

**Authors:** Mengru Zhu, Junhao Xia, Jia Liu, Wei Zou, Xin Guan, Lizhi Wang, Yichen Wang, Bing Wang, Fengya Wang, Qingwen Zhang, Keman He, Lukuan Liu, Jing Liu

**Affiliations:** a Stem Cell Clinical Research Center, The First Affiliated Hospital of Dalian Medical University, Dalian City, Liaoning Province, China; b Liaoning Key Laboratory of Frontier Technology of Stem Cell and Precision Medicine, Dalian Innovation Institute of Stem Cell and Precision Medicine, Dalian, 116011, China; c Department of Plastic Surgery, The First Affiliated Hospital of Dalian Medical University, 222 Zhongshan Road, Dalian, 116011, China; d Department of Radiation Oncology, The First Affiliated Hospital of Dalian Medical University, 222 Zhongshan Road, Dalian, 116011, China

**Keywords:** Nanovesicles, Mitochondrial dysfunction, Mitochondrial homeostasis, Radiation-induced skin injury, DNA repair, Mitophagy

## Abstract

Radiation-induced skin injury (RSI) is characterized by persistent mitochondrial dysfunction and compromised DNA repair mechanisms, posing significant challenges for clinical management. To address this, we engineered mitochondria-enriched nanovesicles (NVs) derived from human umbilical cord mesenchymal stem cells (hUMSCs), designed to deliver bioactive mitochondrial components to irradiated skin tissues. Using established in vitro and in vivo models of X-ray-induced RSI, we demonstrated efficient NV internalization into epidermal and dermal cells, leading to restoration of mitochondrial ultrastructure and metabolic function, attenuation of reactive oxygen species (ROS), and facilitation of DNA damage repair. Data-independent acquisition (DIA) proteomic profiling further indicated that NVs significantly upregulated key DNA repair proteins (including POLD3, POLE4, RFC1, and ERCC6), which were downregulated after irradiation, and activated the PINK1-Parkin mitophagy pathway. Additionally, NVs restored mitochondrial dynamics by suppressing DRP1-mediated fission and enhancing MFN1/2-dependent fusion, collectively promoting cellular homeostasis. These findings support the development of a cell-free, mitochondria-based nanotherapeutic strategy that concurrently targets DNA repair and mitochondrial quality control, presenting a scalable and promising treatment for RSI and potentially other radiation-induced disorders.

## Graphical abstract

**Figure:**
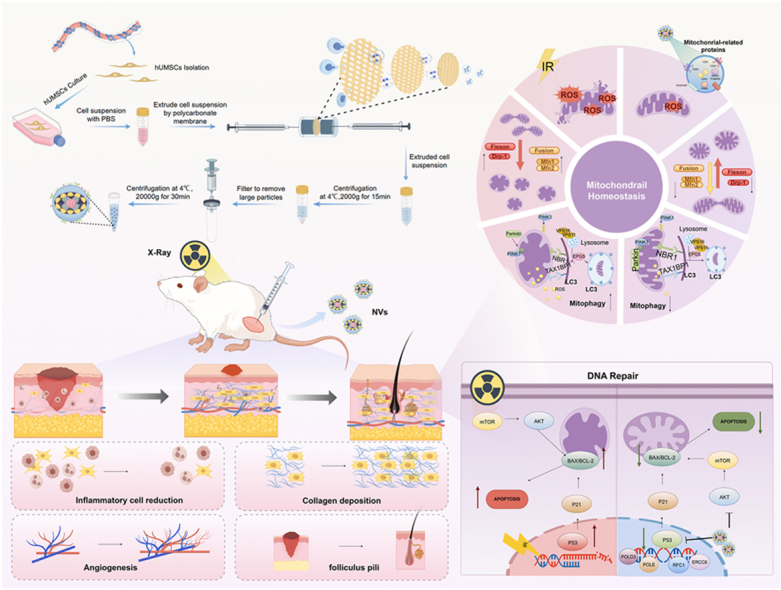

## 1 Introduction

Radiation-induced skin injury(RSI)constitutes an inflammatory injury to the skin and mucous membranes resulting from exposure to various forms of ionizing radiation. It represents a significant adverse effect of radiation therapy, manifesting in up to 95 % of patients undergoing such treatment [1]. This collateral damage initially presents as acute radiation dermatitis (ARD), which emerges within days to months post-radiotherapy and is typified by erythema and desquamation [2]. Patients who develop moderate to severe acute radiation dermatitis that is not promptly treated in its early stages are believed to have an increased risk of irreversible late side effects, such as capillary dilation and fibrosis, in the months to years following radiotherapy (RT) [3]. Nevertheless, there is no universally accepted standard for the management of acute radiation dermatitis, and existing guidelines exhibit considerable variability [4]. Current clinical strategies, including recommended interventions such as the use of modern dressings and topical corticosteroids, remain predominantly focused on symptom management. These therapies are associated with a range of adverse effects, and their limited efficacy and variable therapeutic outcomes highlight the critical need for the development of novel therapeutic strategies [5].

Recent research has demonstrated that mitochondria constitute the principal cytoplasmic target of ionizing radiation and serve as an early indicator of cellular damage [6]. Ionizing radiation has the capacity to directly compromise mitochondrial membrane integrity, provoke the excessive accumulation of reactive oxygen species (ROS), and subsequently initiate DNA damage, energy metabolism disorders, and apoptosis. Concurrently, mitochondrial quality control (MQC) mechanisms, such as mitophagy, play a crucial regulatory role in preserving mitochondrial homeostasis and inhibiting apoptosis [7,8]. Consequently, strategies aimed at repairing or regulating mitochondrial function are regarded as effective approaches for alleviating radiation-induced skin damage.

In exploring such interventions, stem cell therapy has attracted considerable attention as a means of repairing radiation-induced skin damage [9]. Among them, human umbilical cord derived stem cells (hUMSCs) have demonstrated significant regenerative capacity by promoting neovascularization, re-epithelialization, and overall wound healing [10]. However, stem cell therapy faces challenges in terms of cell safety and passage limitations during clinical translation. Importantly, accumulating evidence suggests that the beneficial effects of hUMSCs are largely mediated through paracrine mechanisms, particularly via extracellular vesicles (EVs) [11]. hUMSC-derived EVs have been shown to attenuate oxidative stress, reduce DNA damage, and improve cellular repair in irradiated tissues, mainly through the activation of NRF2 signaling pathways and suppression of cellular senescence [12]. However, their clinical translation is limited by low production yield [13], heterogeneity in size and cargo [14], and the uncontrollable nature of their biogenesis [15], which heavily depends on cellular endocytic and exocytic processes. These factors lead to significant variability in therapeutic efficacy and hinder large-scale, standardized production. Moreover, the uptake efficiency and biodistribution of EVs are inconsistent across target tissues, further compromising their therapeutic consistency. To overcome these challenges, cell membrane-derived nanovesicles (NVs) have been developed as EV mimetics. NVs can be rapidly fabricated in large quantities via mechanical extrusion or sonication [16,17], allowing for enhanced control over vesicle composition, reproducibility, and functional delivery, thereby offering a more scalable and effective alternative for clinical applications [18,19].

Recent studies have demonstrated that NVs derived from stem cells possess significant therapeutic potential in promoting cell proliferation, migration, and angiogenesis, as well as in facilitating wound healing [20]. Notably, NVs not only retain essential signaling molecules from their parent cells but also encapsulate functional mitochondrial components, thereby enabling the modulation of mitochondrial activity in recipient cells [21]. This mitochondria-targeting capacity is particularly relevant in the treatment of RSI, where mitochondrial dysfunction plays a central pathological role. While previous studies have preliminarily suggested the efficacy of NVs in cutaneous wound repair, a comprehensive analysis of the mitochondrial protein cargo within these NVs, and their functional implications remains lacking.

Considering the pivotal role of mitochondrial dysfunction in RSI, we propose and validate an innovative therapeutic approach utilizing hUMSC derived NVs enriched with mitochondrial proteins (mitochondria-enriched NVs, ME-NVs) for the treatment of RSI. Through proteomic analysis, we identified and functionally enriched 638 mitochondrial-related proteins in NVs, which are intricately linked to oxidative phosphorylation, mitochondrial dynamics, and autophagy. Furthermore, our findings demonstrate that these NVs can sustain mitochondrial homeostasis and mitigate radiation-induced apoptosis by modulating the mitotic mechanism mediated by PINK1/Parkin and the equilibrium between mitochondrial fusion and fission. Additionally, data-independent acquisition (DIA) mass spectrometry results revealed that NVs can enhance the expression of DNA repair proteins, including POLD3, RFC1, and ERCC6, thereby augmenting cellular repair capacity and genomic stability. This study not only addresses an unmet clinical need but also lays the groundwork for the translational development of next-generation nanovesicle-based therapeutics in the field of radiation medicine.

## 2 Materials and methods

### 2.1 Patients and ethics approval

The human umbilical cord mesenchymal stem cells (hUMSCs) applied in this research were obtained in accordance with the ethical standards outlined in the Declaration of Helsinki. Prior informed consent was secured from the pregnant donors and their families. The study protocol received ethical approval from the Ethics Committee of the First Hospital of Dalian Medical University (Approval No.: PJ-KS-KY-2023-97).

### 2.2 Cell culture

For hUMSCs culture, the AMMS®MSC Kit 2.0 (Tongli Haiyuan, Beijing, China) was utilized to provide essential nutrients.Human skin fibroblasts (HSF), human keratinocyte cell line (HaCaT) were acquired from Zhong Qiao Xin Zhao Biotechnology (Shanghai, China). Both HaCaT and HSF cells were maintained in high-glucose DMEM (Zhong Qiao Xin Zhao) supplemented with 10 % fetal bovine serum (FBS, Gibco) and 1 % penicillin-streptomycin (OriCell®). All cell lines were incubated under standard sterile conditions at 37 °C in a humidified atmosphere containing 5 % CO_2_ and passed once the cells reached 80–90 % confluence.

### 2.3 Preparation of hUMSCs -derived NVs (NVs) and natural small extracellular vesicles (sEVs)

The hUMSCs -derived NVs (NVs) and natural small extracellular vesicles (sEVs) were prepared. For simplicity, the term ‘NV’ is used in the figures to represent NVs, and ‘EV’ is used to represent sEVs. The hUMSCs were rinsed with PBS and resuspended for counting, adjusting the cell density to 1 × 10^6^ cells/mL. To create NVs, the suspension was extruded through 10 μm, 5 μm, and 1 μm polycarbonate membranes, each step repeated 7 times. The filtrate underwent centrifugation at 2000×*g* for 15 min to remove large fragments. The supernatant was filtered through a 0.22 μm filter and centrifuged at 20,000×*g* for 30 min. The final pellet was reconstituted in PBS and stored at −80 °C for later use as NVs.

Under reaching 70–80 % confluence, the conditioned medium (CM) was harvested following a 48-h incubation. Isolation of sEVs were performed using multi-step centrifugation procedure from CM. Briefly, CM was centrifuged at 1000×*g* for 10 min to remove cells. Subsequent centrifugations at 2000×*g* for 20 min and 10,000×*g* for 30 min were carried out to eliminate cell debris and large vesicles. The final supernatant was then filtered through a 0.22 μm filter and subjected to ultracentrifuged at 100,000×*g* for 120 min (Beckman Coulter Optima XPN-100, SW41Ti rotor). The obtained pellet was washed in sterile phosphate-buffered saline (PBS) and ultracentrifuged under the same conditions. the final pellet, containing purified sEVs, was resuspended in PBS and stored at −80 °C for subsequent experiments.

### 2.4 Characterization of NVs

The particle size and zeta potential were determined using nanoparticle tracking analysis (NTA) using the ZetaView® (PMX-120, Particle Metrix). Morphology of the NV was observed by transmission electron microscope (TEM). Western blotting was used to measure the protein composition. Total proteins from NV were extracted using RIPA lysis buffer (89901, Thermo Fisher Scientific) supplemented with protease and phosphatase inhibitors (78430 and 78420, Thermo Fisher Scientific). The average protein concentration was measured using the Bicinchoninic acid (BCA) protein assay kit (P0011, Beyotime). Equal amounts of protein samples were subjected to electrophoresis on a 4–12 % Bis-Tris gel (MA0463, MeilunBio), using a three-color pre-stained protein standard (AG11919, Accurate Biology) as a molecular weight marker. The separated proteins were then transferred to a 0.2 μm polyvinylidene fluoride (PVDF) membrane (Millipore) for Western blot analysis. The PVDF membrane was incubated with primary antibodies overnight at 4 °C. Subsequently it was incubated with the corresponding HRP-conjugated secondary antibodies at room temperature for 2 h. The membrane was washed three times with tris buffered saline with tween 20 (TBST), and then developed using an enhanced chemiluminescence (ECL) chemiluminescent reagent. Signal visualization was performed using the Champ Chemi 610 Plus imaging system. The primary antibodies included CD9 (ab236630), CD63 (ab134045), HSP70 (ab181606), GAPDH (ab181602). The secondary antibodies included: Goat anti-Rabbit IgG H&L (HRP) (ab6721), Goat anti-Mouse IgG H&L(HRP) (ab6789), all from Abcam, 1:1000.

### 2.5 Endocytosis of the NVs

NVs were labeled with PKH26 (MIDI26, Sigma-Aldrich) and incubated for 5 min at room temperature. The labeling reaction was terminated by adding an equal volume of 1 % bovine serum albumin (BSA). Excess dye was removed by washing the NVs three times with PBS using ultracentrifugation at 100,000×*g* for 70 min. HaCaT and HSF cells were seeded in confocal microscopy dishes (1.5 × 10^3^ cells each) and left to adhere overnight.The medium was then replaced with high-glucose DMEM containing PKH26-labeled NVs, and cells were incubated for 2 h. After washing, cells were stained with 1 μM Calcein AM for the cytoplasm and Hoechst 33342 for the nuclei, and imaged by laser confocal system. Successful NV uptake was indicated by red fluorescence in the cytoplasm. PKH26-labeled NVs showed red fluorescence, Calcein AM stained the cytoplasm green, and Hoechst 33342 marked the nuclei cyan. Quantification of NV uptake rate was performed by calculating the proportion of PKH26-positive cells relative to the total number of DAPI-stained nuclei per field of view. Three non-overlapping fields were analyzed per sample, and the average value was taken as one biological replicate. Each group contained three independent replicates.Results are presented as mean ± SEM. Statistical analysis was conducted using GraphPad Prism software(v9.5.1).

### 2.6 Wound healing assay

A scratch was made using a sterile pipette tip in confluent HSF monolayers. After washing, serum-free medium was added. Images were taken at 0, 18, 24, and 48 h post-irradiation using an inverted microscope. Wound closure percentage was calculated.

### 2.7 Intracellular reactive oxygen species (ROS) detection

Intracellular ROS levels were assessed using the DCFH-DA kit (S0033M, Beyotime, China) as per instructions. HaCaT or HSF cells were grown in 6-well plates to 70–80 % confluence, then treated with NV (10 μg/μl) 2 h before X-ray exposure. After 24 h, cells were incubated with 10 μM DCFH-DA in serum-free medium at 37 °C for 20 min in the dark, followed by three PBS washes. DCF fluorescence, indicating ROS levels, was measured via fluorescence microscopy or flow cytometry (excitation: 488 nm, emission: 525 nm) and analyzed using FlowJo software (v10.8.1)

### 2.8 Mitochondrial membrane potential (ΔΨm) detection

Mitochondrial membrane potential (ΔΨm) of HaCaT and HSF cells was assessed using Tetramethylrhodamine methyl ester (TMRM, I34361, Thermo Fisher Scientific), a cell-permeant fluorescent dye that accumulates in active mitochondria. 24 h after X-ray radiation, cells were seeded in 6-well plates and cultured until they reached 70 % confluence. Following experimental treatments, cells were incubated with 100 nM TMRM diluted in serum-free culture medium at 37 °C for 30 min in the dark. After staining, cells were washed twice with phosphate-buffered saline (PBS) to remove excess dye. Fluorescence was analyzed using flow cytometer (SH800, Sony). The average fluorescence intensity was quantified using FlowJo software (v10.8.1). A reduction in TMRM fluorescence indicates depolarization of the mitochondrial membrane.

### 2.9 Mitochondrial imaging

Cells were seeded on confocal dishes and stained with MitoTracker Deep Red FM (100 nM, Thermo Fisher) and LysoTracker Green DND-26 (75 nM, Thermo Fisher) in serum-free medium at 37 °C for 30 min. After washing with PBS, super-resolution images were performed by Nikon N-SIM 5.0 Super-Resolution Microscope System(Ex:560 nm, collected: 570–640 nm). Skeleton analysis and quantification of mitochondrial morphology were analyzed using the Mitochondrial Analyzer plugin in Image J. Confocal images were recorded with FV1000 (Olympus) with a 100 × /NA 1.44 oil immersion objective lens(Ex:543 nm, collected: 560–660 nm) [22,23]. Mitochondria–lysosome colocalization was quantified by Pearson's correlation coefficient, calculated with the built-in colocalization analysis module of OLYMPUS FLUOVIEW Ver. 3.0.

### 2.10 Apoptosis detection

The Annexin V-FITC Apoptosis Detection Kit (C1062M, Beyotime) was used to measure the apoptosis rate of HaCaT and HSF cells. Added dye to each group according to the instructions and collected cells after a certain period of intervention. Added binding buffer to resuspend cells after centrifugation, stained with AnnexinV-FITC and PI staining solution for 30min and then detected by flow cytometer (SH800, Sony). Annexin V-positive and PI-negative cells (Annexin V-FITC+/PI−) were considered early apoptotic cells, while Annexin V/PI double-positive cells (Annexin V-FITC+/PI+) were considered late apoptotic cells.

### 2.11 Hemolysis assay

To evaluate NV's hemocompatibility, a hemolysis assay was performed using blood from three healthy male SD rats. Blood was collected in sodium citrate tubes, mixed, and centrifuged at 3000 rpm for 15 min at 4 °C. The supernatant was removed, and the RBC pellet was washed with saline and resuspended to a 2 % RBC solution. NV was added at 10 μg/mL and 100 μg/mL concentrations. PBS and distilled water served as negative and positive controls. After a 1-h incubation at 37 °C, the mixtures were centrifuged, and the supernatant's absorbance was measured at 540 nm. The hemolysis ratio was calculated using the formula: Hemolysis ratio (%) = (ODsample − ODnegative)/(ODpositive − ODnegative) × 100 %. Values < 5 % confirmed good hemocompatibility.

### 2.12 Cell viability assay

Cell viability was assessed by performing a CCK-8 assay. HaCaT or HSF cells were seeded in a 96-well plate at a density of 1 × 10^3^ cells per well. The cells were then treated with various experimental conditions, including control, X-ray irradiation(4, 8, 12 and 16 Gy) and NV (2.5, 5, 10, 15, 20, 40 and 60 μg/mL), at different time points (24 h, 48 h and 72 h). The cells were incubated with 100 μL of 10 % CCK-8 solution, diluted in medium without fetal bovine serum (FBS) for 1 h. Following incubation, the absorbance of each well was measured at 450 nm using a microplate reader (Thermo Scientific, Multiskan MK3). The cell viability percentages were calculated relative to the control group.

### 2.13 Animal experiment

SD rats of 6–8 weeks (200 g) were obtained from Liaoning Changsheng Biotechnology Co., Ltd. All animal experiments adhered to the NIH Guide for the Care and Use of Laboratory Animals and were conducted according to the protocol approved by the Institutional Animal Care and Use Committee of Dalian Medical University (Ethics Approval Number: AEE24145). The feeding conditions were set at a temperature of 25 ± 2 °C, a humidity of 50 %, a regular light cycle (12 h light/dark cycle), standard feedstuff food, and purified water. 18 rats were divided into 3 groups randomly (6 per group) as follows: 1) Negative control group(NC). 2) X-ray irradiation group(IR). 3) NV treatment group(NV). Rats were acclimatized for one week and weighed one day prior to the experiment. After anesthetizing with 50 mg/kg pentobarbital, the fur on the right dorsal hip was shaved. Rats in the IR and NV groups were placed on a linear accelerator (Elekta Axesse™, Sweden, 6 MeV X-ray), with non-irradiated areas shielded by lead plates. The skin was irradiated with a 3 cm × 3 cm electron beam at a dose rate of 600 cGy/min, with a total dose of 35 Gy.

### 2.14 Administration of NVs in the rat RSI model

Treatment was initiated upon full establishment of radiation-induced wounds (day 9), with tissue collection performed on day 28. ME-NVs were diluted in sterile saline to a final working concentration of 10 μg/mL immediately prior to application. Each treatment consisted of an intradermal multipoint injection totaling 1.0 mL (equivalent to 10 μg ME-NVs), administered across the wound bed and peripheral margins using a microsyringe. The needle was inserted at an approximate angle of 10°to a depth of ∼2 mm. Nine injection sites were distributed uniformly at intervals of approximately 1 cm, with 0.10 mL delivered per site. An additional volume of ∼0.10 mL was prepared to compensate for potential dead volume and procedural loss. Administrations were repeated every three days, culminating in a total of five treatments and a cumulative dose of 50 μg ME-NVs per rat. Animals in the irradiation control (IR) group received equivalent volumes of sterile saline following the same injection protocol. All procedures were carried out under aseptic conditions. Post-administration monitoring revealed no instances of localized bleeding, fluid leakage, or behavioral distress, indicating an absence of procedure-related adverse effects.

### 2.15 Histopathology and immunohistochemistry detection

After 6 rats per group were sacrificed, the skin tissues were collected and fixed with formaldehyde (4 %). The tissues were then made into paraffin and stained with H&E for histopathological analysis. Masson's trichrome staining was performed using a commercial kit (Servicebio, China) according to the manufacturer's instructions. And then, TUNEL staining was utilized to analyze the apoptosis of skin tissue in 3 rats per group. Finally, the images were captured by the inverted fluorescent microscope. For CD31 (66065-2-1g, Proteintech) and α-SMA (19245S, Cell Signaling Technology) immunohistochemical staining, the average optical density values were used to characterize vascularization in regenerated tissue. Quantitative analysis was performed using ImageJ/Fiji v1.54. Images were split into individual channels for CD31 (green) and α-SMA (red), background was subtracted, and signals were converted to 8-bit grayscale. The average optical density (AOD) was computed as integrated optical density (IOD) divided by area (AOD = IOD/area) to quantify signal intensity. Three random non-overlapping fields (200 × ) per animal were analyzed, and the mean AOD was used as one data point (n = 3). Values were normalized to the control mean and expressed as relative expression. Data are shown as mean ± SEM, and statistical analysis was performed with GraphPad Prism 9.5.1.

### 2.16 Label-free proteomic analysis of NVs

Proteins were extracted from purified NVs (10^7^/sample) using RIPA buffer with protease inhibitors and quantified by BCA assay. After reduction with DTT and alkylation with iodoacetamide, proteins were digested with trypsin overnight at 37 °C. Peptides were desalted and analyzed bySCIEX M5 MicroLC microliter liquid chromatograph coupled with a Triple-TOF 5600+ mass spectromete. MS data were acquired in data-dependent acquisition (DDA) mode and processed with MaxQuant software using the UniProt human database. Data retrieval and quantitative analysis: the obtained raw data were searched using MaxQuant (v1.6.1.0) against the Swiss-Prot human protein database (UniProt Proteome, release 20220128). Gene Ontology (GO) term enrichment analysis and protein-protein network analysis were based on DAVID 2021 (Dec. 2021) and STRING (version 11.5) databases. The mitochondrial protein reference set used for intersection and enrichment analyses was derived from MitoCarta 3.0 (Broad Institute, maintained by the Mootha Laboratory) [24].

### 2.17 Quantitative proteomics by DIA-MS of HSF

To characterize global proteomic changes in response to radiation and nanovesicle (NV) treatment, we performed data-independent acquisition mass spectrometry (DIA-MS) analysis using the Orbitrap Astral™ mass spectrometer (Thermo Fisher Scientific), integrated with a streamlined sample preparation workflow. HSF were harvested 48 h post-irradiation, lysed in TEABC buffer containing RapiGest, TCEP, and CAA, and digested with trypsin overnight. Peptides were desalted and analyzed via nanoLC-MS/MS on a PepMap C18 column with a 120-min gradient. DIA data were acquired with high-resolution MS1 and MS2 scans, using variable isolation windows across 400–1000 *m*/*z*. A hybrid spectral library was generated from DDA and libDIA data. Protein identification and quantification were conducted in Spectronaut with a 1 % FDR cutoff. Over 2000 proteins were quantified per sample with high reproducibility (Pearson's r > 0.95). Differentially expressed proteins (DEPs) were identified based on fold change and adjusted P-values, and subjected to GO and Kyoto Encyclopedia of Genes and Genomes (KEGG) enrichment analyses.

### 2.18 Statistical analysis

The data of histopathology and immunohistochemistry detection are presented as mean ± standard error of the mean(SEM). All data are presented as mean ± standard deviation (SD) of greater than or equal to three separate experiments. Statistical analyses were performed using GraphPad Prism 9.5.1 (GraphPad Software, San Diego, CA, USA). Two-tailed unpaired *t*-test, one-way ANOVA, and two-way ANOVA were used. Bonferroni’ s correction for multiple comparisons was used to calculate adjusted p value when appropriate. Statistical significance was considered as p < 0.05.

## 3 Results and discussion

### 3.1 Fabrication, characterization and analysis of mitochondrial protein-enriched hUMSC-NVs

**Fig. 1:**
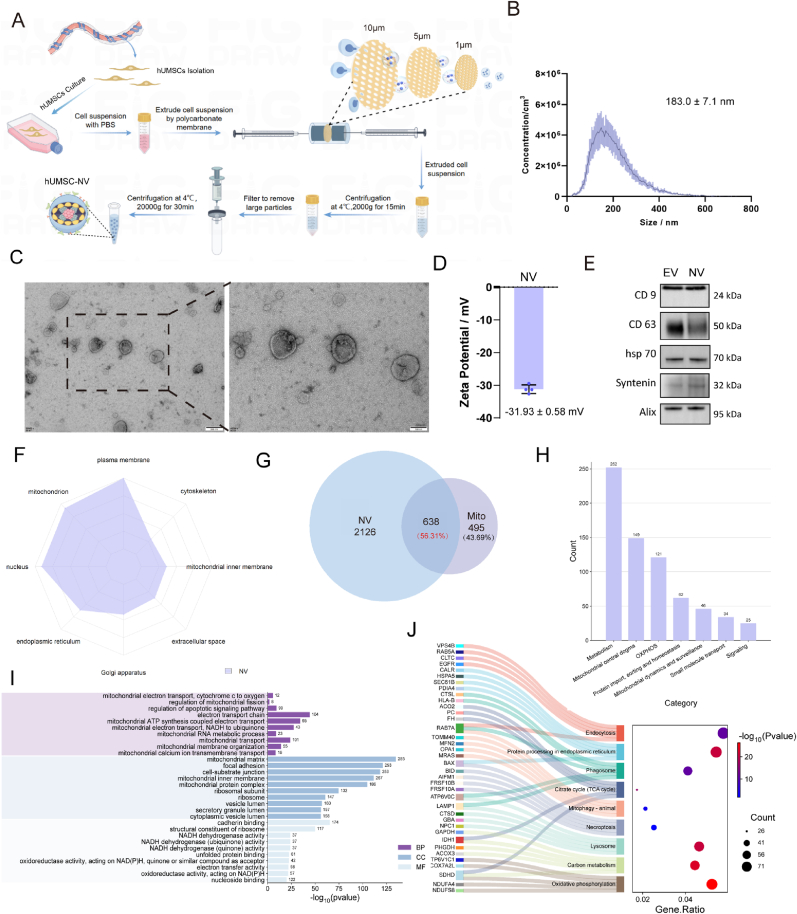
Fabrication, characterization and analysis of mitochondrial Protein-Enriched hUMSC-NVs. A) Flowchart of the isolation of NVs. (Created by Figdraw, ID: WIWSS4a36a). B) Nanoparticle tracking analysis (NTA) of the range of particle size distribution of NVs. C) Transmission electron microscopy (TEM) images (scale bars, 200 nm) and magnification (scale bars, 100 nm) of NVs. D) Zeta potentials of NVs. E) Measurements of the EV markers (CD9, CD63, hsp70, Syntenin and Alix). F) Subcellular localization of NV-proteins. G) Venn analysis of all proteins from NVs and MitoCarta database. H) Functional classification of Mitochondrial Proteins in NVs. I) Gene Ontology (GO) analysis results of total proteins in NVs. BP, CC, and MF represent the three main categories of GO enrichment analysis: biological process (BP), cellular component (CC), and molecular function (MF). J) Sankey-Bubble diagram depicting highly enriched KEGG pathways and the hub genes within each pathway.

To fabricate hUMSCs-NVs, hUMSCs were sequentially extruded through several polycarbonate membranes with a graded reduction in pore size (Fig. 1A). This process promoted vesicle size reduction and efficient encapsulation of intracellular proteins, yielding stable and biologically functional NVs. The resulting vesicles exhibited a mean diameter of 183.0 ± 7.1 nm (Fig. 1B). This method fragments cellular membranes via shear force, enabling the self-assembly of the fragments into closed cup-shaped vesicles (Fig. 1C). The surface charge, measured by zeta potential, was −31.93 ± 0.58 mV (Fig. 1D), consistent with a negatively charged surface conducive to cellular internalization. Furthermore, standard small extracellular vesicle (sEV) protein markers were reliably detected (Fig. 1E). Notably, the protein production yield of NVs was 15.2 times higher than that of sEVs (Table S1). These results indicate the successful fabrication of hUMSC-derived NVs resemble natural sEVs in terms of features and have a markedly enhanced protein yield. Further proteomic characterization was conducted to identify the bioactive molecules underlying their functional efficacy.

Proteins are crucial for NVs function. The proteome of hUMSCs-NVs was used to identify key bioactive proteins that underlie their therapeutic potential. Protein function is typically closely linked to subcellular localization. NVs carry diverse proteins derived from multiple subcellular origins, including the plasma membrane, mitochondria, and endoplasmic reticulum (Fig. 1F). Although plasma membrane proteins constituted the largest group, we focused on mitochondrial components due to their functional relevance. Given the context of radiation-induced skin injury, where mitochondrial dysfunction plays central pathological roles, we prioritized the investigation of mitochondrial proteins. Among the identified proteins, 638 were mitochondrial in origin, representing 56.31 % of the reference mitochondrial proteome defined by MitoCarta 3.0 database [[24], [25], [26]], indicating substantial incorporation of mitochondrial contents during vesicle formation via membrane reassembly (Fig. 1G). Distribution shows that although the NVs contain proteins from diverse cellular compartments, mitochondrial proteins constitute the most abundant functional group among the shared fraction (Fig. 1H). To further investigate the potential biological roles of these shared proteins, functional enrichment analysis was specifically performed on this subset. GO analysis revealed a pronounced mitochondrial signature in the NV proteome (Fig. 1I). The most significantly enriched Biological Process (BP) terms were associated with mitochondrial electron transport and ATP synthesis coupled to electron transport. These findings provide molecular evidence supporting the potential of NVs to enhance oxidative phosphorylation, sustain mitochondrial membrane potential (ΔΨm), and alleviate oxidative stress in recipient cells. Enrichment of terms such as'regulation of mitochondrial fission’ and ‘regulation of apoptotic signaling pathway'further suggests that NV cargo may modulate the DRP1-MFN/OPA1 axis, which is critical for mitochondrial dynamics and cell survival under stress conditions. Cellular Component (CC) terms were predominantly localized to the mitochondrial inner membrane, mitochondrial matrix, and mitochondrial protein complexes. Molecular Function (MF) enrichment highlighted terms were related to NADH dehydrogenase and electron-transfer oxidoreductase activities. The concurrent enrichment of focal adhesion and cadherin-binding terms implies a potential role in modulating adhesion and migration behaviors, which may act synergistically with improved bioenergetic capacity. A sankey diagram based on KEGG analysis was employed to depict the functional enrichment and representative NV-associated proteins (Fig. 1J). Several key regulators of mitochondrial dynamics and selective autophagy were identified. These included Optic Atrophy 1(OPA1) and Mitofusin 2 (MFN2), which are crucial for mitochondrial fusion and integrity. *BAX*, involved in membrane permeabilization and mitophagy. VDAC1, important for metabolite transport and stress signaling. Given the central role of mitochondrial dysfunction-marked by respiratory deficit, ROS overproduction, and energetic failure in radiation-induced tissue damage, the mitochondrial protein enrichment within NVs suggests a compelling mechanism for functional recovery in recipient cells. These findings indicate that the primary therapeutic action of hUMSC-NVs stems from their mitochondrial regulatory activity, justifying their designation as mitochondria-enriched nanovesicles (ME-NVs).

### 3.2 NV treatment effectively alleviates X-ray-induced acute radiation skin injury in a rat model

**Fig. 2:**
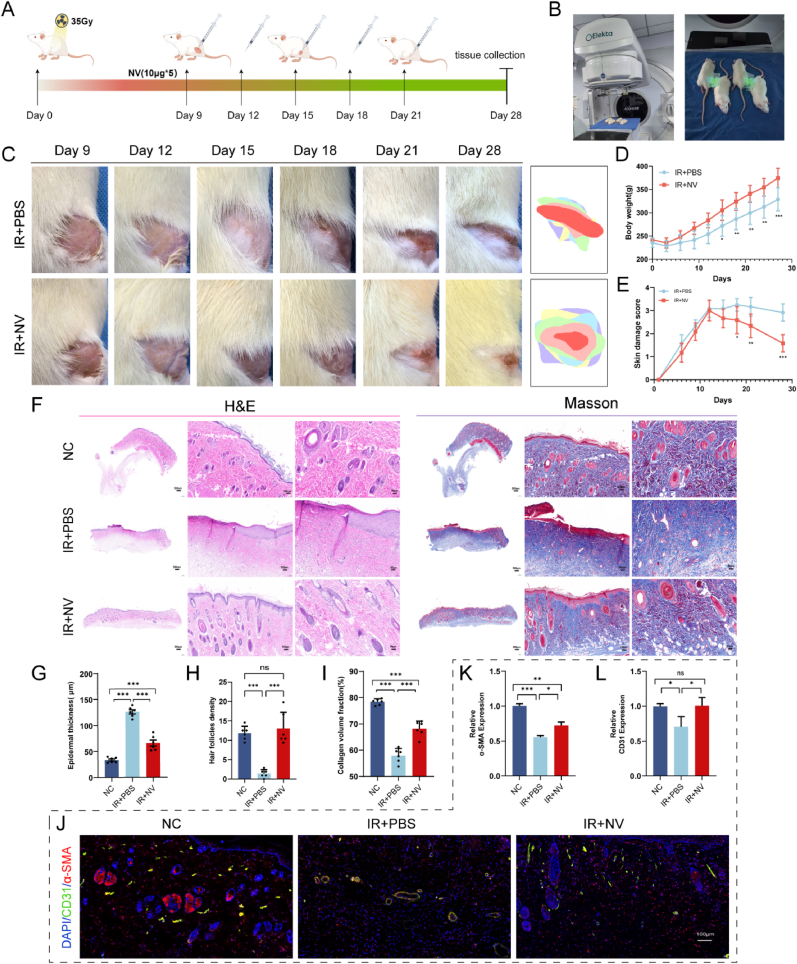
NV treatment effectively alleviates X-ray-induced acute radiation skin injury in a rat model. A) Schematic description of the establishment and treatment of the ARD model in vitro(Created by Figdraw, ID:OIUWT68076). B) Elekta Axesse linear accelerator and operation process in the Rat ARD model.C) Representative graphs of wound healing after radiation at six-time points. D) The change of body weight after radiation(n = 6). E) Skin samples were visually scored for radiation injury(n = 6). F) Representative images of H&E staining and Masson's trichrome staining of wound tissues on day 28 after radiation. G-I) Epithelial thickness analysis, Hair follicle density and collagen deposition fractionon of 3 groups on day 28 after radiation(n = 6). J-L) Immunofluorescence staining of wound tissue sections on day 28 after radiation. by labeling CD31 (green) and α-smooth muscle actin (α-SMA) (red) and comparing the expression levels of CD31 and α-SMA in each group (n = 3). The results are expressed as the mean ± SEM. Scale bars: left, 500 μm, middle, 100 μm, right, 50 μm ns, not significant, ∗p < 0.05, ∗∗p < 0.01 and ∗∗∗p < 0.001. (For interpretation of the references to color in this figure legend, the reader is referred to the Web version of this article.)

To evaluate the therapeutic potential and bio-safety of NVs in vivo, we established a rat model of acute radiation-induced skin injury. Rats were exposed to 35 Gy X-ray irradiation on day 0 and started treatment on day 9. NVs or PBS injections were administered every three days, with skin tissue samples harvested on day 28 for comprehensive analysis (Fig. 2A and B). Macroscopic observation of the irradiated skin areas at day 9, 12, 15, 18, 21, and 28 to monitor the wound healing process (Fig. 2C). In the IR + PBS group, the wounds exhibited persistent ulceration, scab formation, and delayed healing throughout the observation period. In contrast, the IR + NV group showed visibly improved skin conditions, with accelerated wound closure and reduced lesion severity. By day 15, the NV-treated rats showed smoother wound surfaces, and by day 28 wound size was significantly reduced compared to the PBS-treated group. After treatment, significant weight gain was observed in the rats compared with the PBS group (Fig. 2D). The skin damage score demonstrates that the wound extent was consistently lower after NV treatment than that in comparison with the PBS group (Fig. 2E). Histopathological analysis was used to assess skin condition and treatment effects (Fig. 2F). H&E staining showed the IR + PBS group had a thicker epidermis (Fig. 2G), crust formation, loss of dermal appendages, and pronounced inflammatory infiltration. While the IR + NVs group had better skin integrity, reduced epidermal thickness, and more hair follicles (Fig. 2H). Masson's trichrome staining indicated disorganized collagen in the IR + PBS group, while better organized and increased collagen in the IR + NVs group (Fig. 2I). Immunofluorescence staining was performed to assess cell activity and neovascularization (Fig. 2J). Compared with the NC group, the IR + PBS group displayed markedly reduced CD31 (Fig. 2K) and α-SMA expression (Fig. 2L), reflecting impaired vascular formation after radiation injury. NV treatment (IR + NVs group) restored both markers, with vessel density nearly comparable to the NC group.

To further assess the bio-safety of NVs, hemocompatibility and systemic toxicity were evaluated. The hemolysis rates of both low concentration (10 μg/mL) and high concentration of NVs (100 μg/mL) were below the accepted safety threshold of 5 %, indicating that the NVs have good hemocompatibility (Fig. S1A). Moreover, H&E staining showed no toxicity in major organs including heart, liver, lungs, and kidneys after injection of NVs solution (Fig. S1B).

### 3.3 NV treatment rescues mitochondrial function and attenuates DNA damage in radiation-induced skin injury

**Fig. 3:**
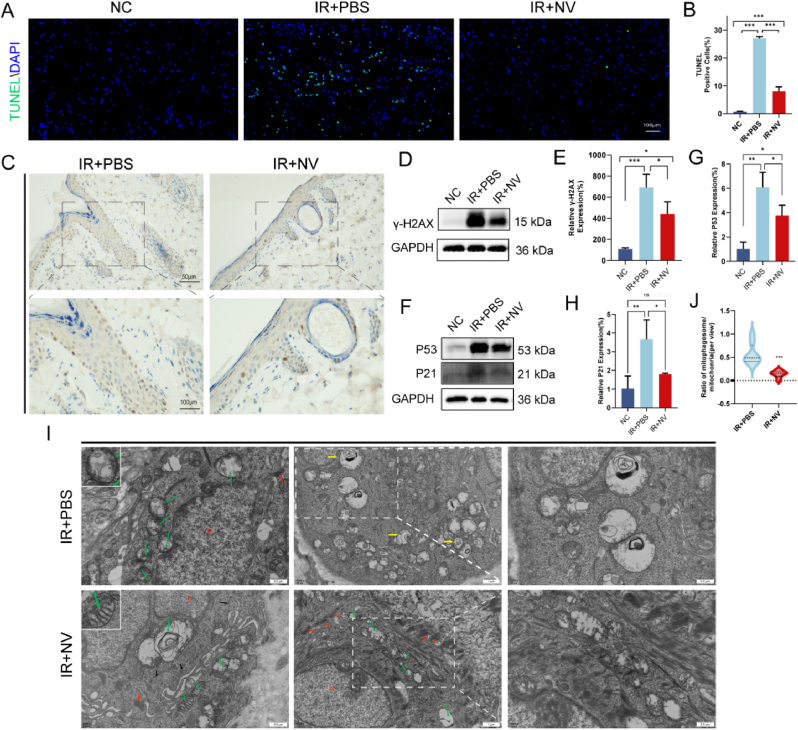
NV Treatment rescues mitochondrial function and attenuates DNA damage in radiation-induced skin injury. A) Fluorescence labeling of TUNEL (FITC filter/green), and nuclei (DAPI filter/blue), scale bars:100 μm. B) Quantitative analysis of TUNEL positive cell. C)γ-H_2_AX immunohistochemistry in skin tissues, and black boxed areas are enlarged. Scale bars: top, 50 μm, Bottom, 100 μm. D) Western blot analysis of expression levels of γ-H_2_AX in each group, GAPDH was used as a loading control. E) Quantification of γ-H_2_AX. F,H) Western blot analysis and quantification of expression levels of P53 and P21 in each group, GAPDH was used as a loading control. I) Transmission electron microscopy of irradiated rats' skin tissues. Green arrows indicate mitochondria, red arrows indicate cell-cell junction structures such as desmosomes, yellow arrows indicate mitophagosomes. Scale bars: left and right, 0.5 μm, middle, 1 μm. J) Violin plot showing the ratio of mitophagosome to mitochondria. The respective number of the vesicles was normalized by observed area(μm^2^). Data are represented as mean ± SD (n = 3). ns, not significant, ∗p < 0.05, ∗∗p < 0.01 and ∗∗∗p < 0.001. (For interpretation of the references to color in this figure legend, the reader is referred to the Web version of this article.)

Having established the efficacy and bio-safety of ME-NVs in a rat model of radiation-induced skin injury, we next sought to explore the underlying mechanisms. For apoptosis analysis, TUNEL staining demonstrated a marked increase in apoptotic cells in the IR + PBS group, consistent with radiation-induced cell death. In contrast, the IR + NVs group exhibited a notable reduction in TUNEL-positive cells, indicating that NV treatment effectively attenuated apoptosis and enhanced cell viability (Fig. 3A and B). Similarly, γ-H2AX immunofluorescence—a sensitive marker of DNA double-strand breaks(DSBs) [6]—showed intense positivity following irradiation but significantly diminished in the NVs-treated group, suggesting improved DNA repair capacity (Fig. 3C). Western blot analysis further corroborated these findings. That confirmed altered expression of proteins related to apoptosis and DNA damage (Fig. 3D and E). Radiation-induced DNA damage activates the protein P53, which plays a pivotal role in maintaining genomic stability by inducing cell cycle arrest, facilitating DNA repair, and triggering apoptosis [27]. Furthermore, P53 can bind to the promoter of P21, leading to its activation [28]. P21 subsequently inhibits cell cycle progression by interacting with cyclin-dependent kinases (CDKs), particularly during the G1 to S phase transition. Statistical analysis of WB data (Fig. 3F and G) revealed that NV treatment reduced the expression levels of P53 and P21 compared to those observed in the IR + PBS group. These results imply that NVs promote tissue regeneration post-irradiation by modulating the p53/p21 pathway, thereby mitigating cell death, reducing senescence, and supporting DNA repair processes. Mitochondrial ultrastructural alterations in rat skin were examined by TEM four weeks after radiation. (Fig. 3I). IR + PBS group exhibited swollen mitochondria with disrupted cristae (marked by green arrows). Additionally, autophagic vacuoles containing fragmented mitochondrial structures were observed (highlighted by yellow arrows), which are characteristic of mitophagy, a process associated with cellular stress and damage. In contrast, the morphology of mitochondria in the IR + NVs group after NV treatment partially restored to normal, exhibited well-defined cristae and intact membranes (marked by green arrows) across all samples. Mitophagy, a selective lysosome-mediated clearance pathway, is essential for maintaining mitochondrial integrity and function. Dysregulated mitophagy leads to the accumulation of damaged mitochondria, exacerbating oxidative and inflammatory stress. [29]. Reduction in autophagic mitochondria was observed, and number of mitophagosome/mitochondrial (per view) also indicated a decrease mitophagy compared to the IR + PBS group (Fig. 3J). Besides, the increased presence of desmosome (marked by red arrows) can enhance the adhesive function and migration of cells under mechanical stress and also regulate signaling pathways involved in changes in cell behavior and tissue morphogenesis [30,31]. This could reflect the positive regulatory effect of NVs therapy on maintaining the structural integrity of skin tissue, aligning with the MF results of NV proteome findings from in vitro experiments. Taken Together, these observations indicate that NV treatment mitigates radiation-induced mitochondrial injury and promotes mitochondrial integrity in vivo.

### 3.4 Dose-Dependent Effects of X-ray exposure on the survival and mitochondrial function of skin cells

**Fig. 4:**
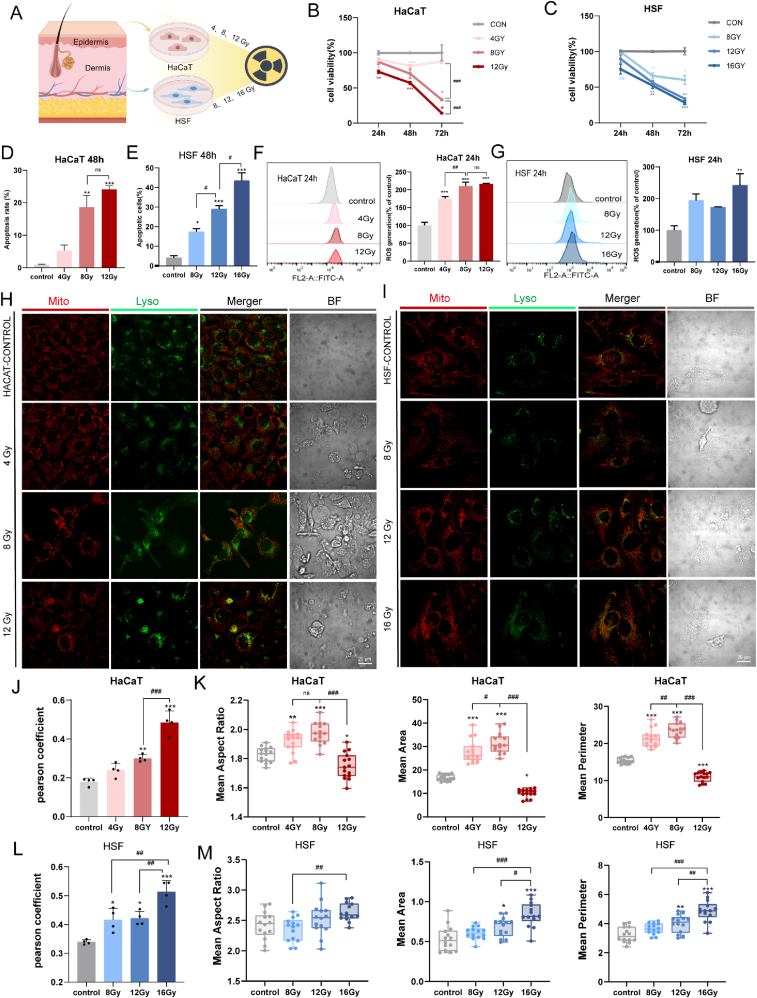
Dose-Dependent Effects of X-ray exposure on the survival and mitochondrial function of skin cells. A) Schematic diagram of the in vitro radiation-induced skin injury model (Created by Figdraw, ID: SYARUfbf13). B,C) Cell viability of HaCaT (left) and HSF (right) was measured at different hours post-irradiation with increasing doses of X-rays(n = 3). D, E) Quantification of total apoptosis rate in HaCaT(D) and HSF (E) at 48 h after increasing doses of X-rays (n = 3). F,G)Flow cytometry analysis and quantification of ROS production in HaCaT(F) and HSF(G) 24h after exposure to increasing dose of X-ray irradiation(n = 3). H,I) Confocal microscopy images of HaCaT(H) and HSF (I) stained with MitoTracker (red) and LysoTracker (green) 24h post-irradiation. Scale bars: 20 μm. BF: bright field. J) Pearson's coefficient for the co-localization of mitochondria with lysosome of HaCaT(n = 14 cells per group). K) Quantification of mitochondrial morphology of HaCaT including mean aspect ratio, mean area, and mean perimeter (n = 3). L) Confocal microscopy images of HSF stained with MitoTracker (red) and LysoTracker (green) 24h post-irradiation. Scale bars: 20 μm. M) Pearson's coefficient for the co-localization of mitochondria with lysosome of HSF(n = 4). M) Quantification of mitochondrial morphology of HSF including mean aspect ratio, mean area and mean perimeter (n = 14 cells per group). P < 0.05, ∗∗P < 0.01, ∗∗∗P < 0.001 vs. control ^#^P < 0.05, ^##^P < 0.01, ^###^P < 0.001 between dose groups. (For interpretation of the references to color in this figure legend, the reader is referred to the Web version of this article.)

The promising outcomes of NVs in vivo prompted us to further validate the therapeutic potential of NVs and elucidate the underlying mechanisms at the cellular level. We next extened our investigation in vitro using human-derived skin cells. To simulate full-thickness skin structure, human keratinocytes (HaCaT) and human skin fibroblasts (HSF) were cultured separately to represent the epidermal and dermal compartments. Cells were exposed to graded doses of X-ray irradiation (HaCaT: 4, 8, 12 Gy. HSF: 8, 12, 16 Gy) to induce radiation-induced injury and establish an in vitro model of acute skin injury (Fig. 4A). Cell viability, as measured by CCK-8 assay, exhibited a dose-dependent decline in both HaCaT and HSF cells following X-ray exposure (Fig. 4B). Compared to the control group (0 Gy), HaCaT exposure to 8 Gy resulted in a moderate but statistically significant reduction in cell viability (Fig. 4B). HSF cells exhibited a sharp decline in viability, with the survival fraction dropping down below 50 % in the 16 Gy group (Fig. 4C). This reveals that high-dose radiation severely impairs cell survival. HaCaT cells exhibited a more pronounced decline in viability than HSF cells at each corresponding dose, further emphasizing the higher radiosensitivity of HaCaT. Annexin V/PI staining combined with flow cytometry revealed a significant increase in both early and late apoptosis with increasing radiation doses at 48h after radiation. HaCaT cells showed no significant difference in apoptosis rates between the 8 Gy and 12 Gy groups, suggesting a plateau effect (Fig. 4D). In contrast, apoptosis in HSF cells increased significantly with higher radiation doses, indicating greater dose sensitivity (Fig. 4E).

Mitochondria are central regulators of apoptosis. As the only extranuclear organelles containing DNA, they are particularly susceptible to radiation-induced injury due to the lack of histone protection [32,33]. The levels of intracellular ROS rose significantly in a dose-dependent manner which directly demonstrated the mitochondrial dysfunction (Fig. 4F and G). Importantly, 8Gy and 12Gy showed no difference in HaCaT, while only 16Gy can cause sever ROS accumulation. To explore the effects of different radiation doses on mitophagy, we further performed the co-localization analysis of mitochondria and lysosomes. Mitochondria were stained with MitoTracker Red, and lysosomes were labeled with LysoTracker Green. As the radiation dose increased, the intensity of green fluorescence exhibited a dose-dependent decrease. Since, high doses of radiation led to a significant dose-dependent decrease in ΔΨm (Fig. 4H and I). The degree of co-localization was quantified using Pearson's correlation coefficient. Both types of skin cells (HACAT and HSF) revealed that a dose-dependent increase in mitochondrial-lysosomal interaction, indicative of enhanced mitophagic flux following irradiation (Fig. 4J–L). The findings underscore the importance of mitophagy in the cellular response to high-dose radiation and are consistent with observations from in vivo experiments. Changes in mitochondrial morphology can impact mitochondrial function, we further use Mitochondria Analyzer to analyze the mitochondrial morphology at 24h post radiation. In HaCaT cells, radiation dose led to a significant increase in aspect ratio, suggesting mitochondrial elongation or fusion. Mitochondrial area and perimeter initially rose at 4–8 Gy, consistent with swelling, but sharply declined at 12 Gy, indicating fragmentation (Fig. 4K). HSF cells showed similar patterns, with aspect ratio, area, and perimeter increasing significantly at higher doses (12–16 Gy), reflecting mitochondrial remodeling or compensatory responses (Fig. 4M).

These findings collectively demonstrate that HaCaT cells exhibit heightened radiosensitivity with early saturation of injury at 8 Gy, whereas HSF cells require higher doses (16 Gy) to reach achieve levels of damage. Therefore, 8 Gy and 16 Gy were selected as optimal radiation doses for HaCaT and HSF cells, respectively, in subsequent experiments to model moderate but distinguishable injury responses in each cell type. Taken together, radiation-induced mitochondrial imbalance in skin cells likely represents an early event and a key amplifier of radiation-induced skin injury.

### 3.5 NVs mitigated radiation induced skin injury of HaCaT and HSF in vitro

**Fig. 5:**
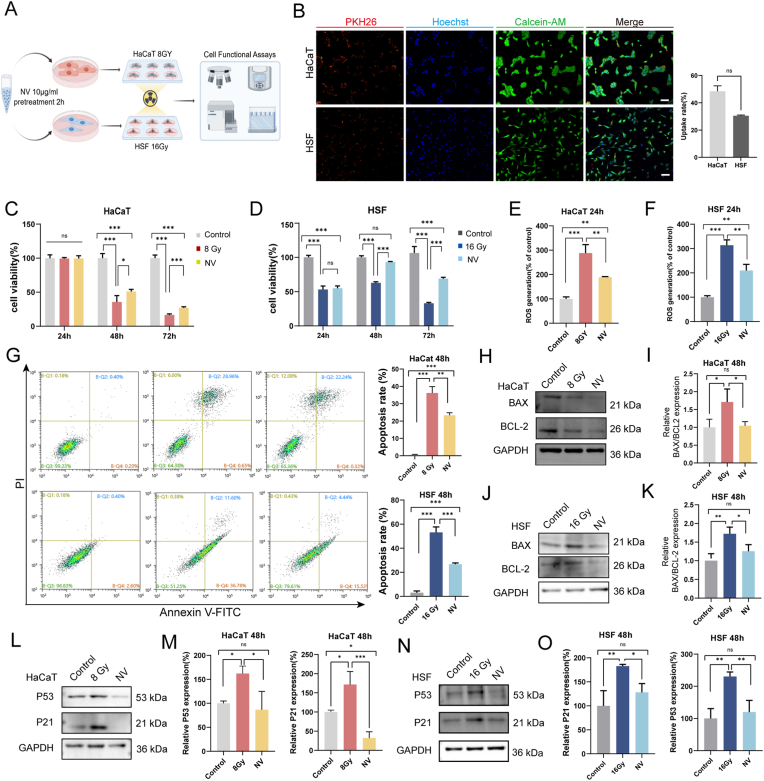
NVs mitigated radiation induced skin injury of HaCaT and HSF in vitro. A) Schematic workflow showing NV pretreatment (10 μg/mL, 2 h) followed by X-ray irradiation (HaCaT: 8 Gy, HSF: 16 Gy), with subsequent functional analyses(Created by Figdraw, ID:YOOWY91917). B) Representative immunofluorescence images of depicting the uptake of PKH26-labeled NVs (red) by HaCaT and HSF cells following a 2-h incubation period. Nuclei are stained with Hoechst (blue) and viable cells are labeled with Calcein-AM(green. The bar graph quantifies the percentage of PKH26-positive cells relative to the total number of DAPI-stained nuclei. Data are presented as mean ± SEM, n = 3. C,D) CCK-8 assay showing NV-mediated rescue of cell viability at 24, 48, and 72 h post-irradiation in (C) HaCaT and (D) HSF, n = 5. E,F) Quantification of intracellular ROS levels (DCF fluorescence) at 24 h post-irradiation in HaCaT (E) and HSF (F), showing significant reduction after NV treatment.(G) Flow cytometry analysis of apoptosis (Annexin V-FITC/PI) at 48 h post-irradiation, with corresponding quantification showing decreased apoptotic cell populations after NV intervention(n = 3). H-K) Western blot analysis and quantification of apoptosis-related proteins BAX and BCL-2 in (H,I) HaCaT and (J,K) HSF at 48 h. L-O) Western blot analysis and quantification of P53 and P21 in (L,M) HaCaT and (N,O) HSF at 48 h. GAPDH was used as a loading control, n = 3. ∗P < 0.05, ∗∗P < 0.01, ∗∗∗P < 0.001. ns, not significant. (For interpretation of the references to color in this figure legend, the reader is referred to the Web version of this article.)

HaCaT and HSF cells were pretreated with NVs for 2 h before radiation, as illustrated in the flow chart (Fig. 5A). NVs exert their biological functions and deliver mitochondrial proteins through internalization by target cells. Using laser confocal quantitative image cytometry, we confirmed that rapid uptake of PKH26-labeled NVs by normal human skin cells. Robust intracellular signal was detectable within 2 h in both HaCaT and HSF (Fig. 5B). The uptake rate in HaCaT was 48.43 ± 6.85 %, modestly higher than in HSF (30.47 ± 1.00 %),indicating efficient and early cellular entry of NVs. Cell survival of HaCaT and HSF was detected by CCK8 assay. NVs has been identified as no cytotoxicity by CCK8 assay, and 10 μg/mL can be the appropriate therapeutic concentrations (Fig. S2A and B). Quantitation results showed NV treatment effectively restored cell viability at 48 h and 72 h, indicating a protective effect against radiation-induced cytotoxicity (Fig. 5C and D). Additionally, scratch wound assay showed that migration was suppressed by irradiation, and NVs enhanced ability to promote skin cell migration (Fig. 3A and B). Excessive production of ROS aggravates radiation damage to the skin. The higher the radiotherapy dose, the more severe the radiation damage to normal tissues, which is a vicious circle. Thus, the ROS clearance after radiation are indispensable procedures for treatment of radioactive skin trauma. Notably, NVs possess antioxidant properties by significantly reducing ROS accumulation in HaCaT and HSF through Flow cytometric analysis at early stage (Fig. 5E and F). Besides, Annexin V/PI staining and flow cytometry analysis (Fig. 5G) reveal NVs suppress radiation-induced apoptosis. That reflected a potential protective role in maintaining cellular integrity. Substantially increase harmful ROS production further triggers apoptosis. ROS have been reported to activate *BAX* through a Bcl-2-suppressible pathway [34,35]. BAX is pro-apoptotic protein whose activation induces mitochondrial outer membrane permeability (MOMP), leading to cytochrome *c* release and subsequent activation of the intrinsic apoptotic pathway [36]. BAX not only acts as a signal for mitophagy, but also promots mitochondrial fragmentation and clearance. BCL2 plays opposing roles in radiation-induced mitochondrial stress, by inhibiting mitochondrial permeability, allowing mitochondria to undergo repair rather than being a target of mitophagy [37].Western blot analysis showed an significantly increase (p < 0.01) in BAX/BCL-2 after x-ray exposure, while NV treatment partially reverses these changes in both HaCaT(Fig. 5H and I) and HSF(Fig. 5J and K) cells, further confirming the anti-apoptotic effects of NVs. Additionally, the role of mitochondria in apoptosis is not limited to their involvement in cell death pathways. They are also central to other forms of cell death, such as pyroptosis, ferroptosis, and necroptosis, highlighting their potential as therapeutic targets. The intersection of these pathways underscores the complexity of mitochondrial involvement in cell death and the potential for therapeutic manipulation [38]. The regulation of apoptosis by NVs and their potential connection to mitochondrial function is an intriguing area of research that warrants further investigation. We next examined whether P53 signaling was influenced in cellular models. Western blot results show that NV treatment notably decreases P53 and P21 expression in both HaCaT (Fig. 5L and M) and HSF (Fig. 5N and O) cells, potentially influencing cell cycle checkpoints and allowing cells more time to repair DNA damage. Radiation-induced p53 activation increased Bax expression, mitochondrial membrane permeability, and triggered the caspase cascade. NV treatment effectively inhibited this p53 over-activation, reducing Bax expression and caspase activity, and inhibiting the apoptosis pathway. Collectively, these results reveal that NV treatment can effectively scavenge ROS at an early stage, enhance skin cell survival following X-ray radiation, promote wound healing by facilitating cell migration, attenuate radiation-induced apoptosis via P53 pathway regulation, and ultimately restored cellular homeostasis.

### 3.6 NV inhibit radiation-induced mitophagy and restore mitochondrial homeostasis

Mitochondria serve as the central hub of cellular aerobic metabolism. Under radiation stress, excessive production of ROS and consequent mitochondrial dysfunction often dictate the fate of cells, driving apoptosis, necrosis, or senescence. Thus, mitochondrial homeostasis is a key determinant of skin cell integrity following injury. Previous studies have established that mitochondrial dynamics and mitophagy collectively maintain this homeostasis. Fission facilitates the isolation and removal of depolarized or damaged mitochondria, while fusion dilutes harmful components. Key molecular players include dynamin-related protein 1 (DRP1) and fission protein 1 (FIS1) in fission, and mitofusin 1/2 (MFN1/2) and optic atrophy 1 (OPA1) in fusion. Disruption of these processes results in aberrant mitochondrial morphology and network organization, impairs interorganellar exchange of genetic and metabolic materials, reduces mitochondrial membrane potential (ΔΨm), and compromises bioenergetic homeostasis. Therefore, we sought to determine whether NV treatment modulates these processes to preserve mitochondrial integrity.Our in vitro and in vivo results demonstrate that ME-NVs restore mitochondrial quality control under radiation stress. Specifically, NVs suppress PINK1/Parkin overactivation and aberrant mitophagy, preventing the loss of functional mitochondria. Concurrently, they correct DRP1/MFN/OPA1-mediated fission-fusion imbalance, thereby restoring ΔΨm and mitochondrial network architecture.

**Fig. 6:**
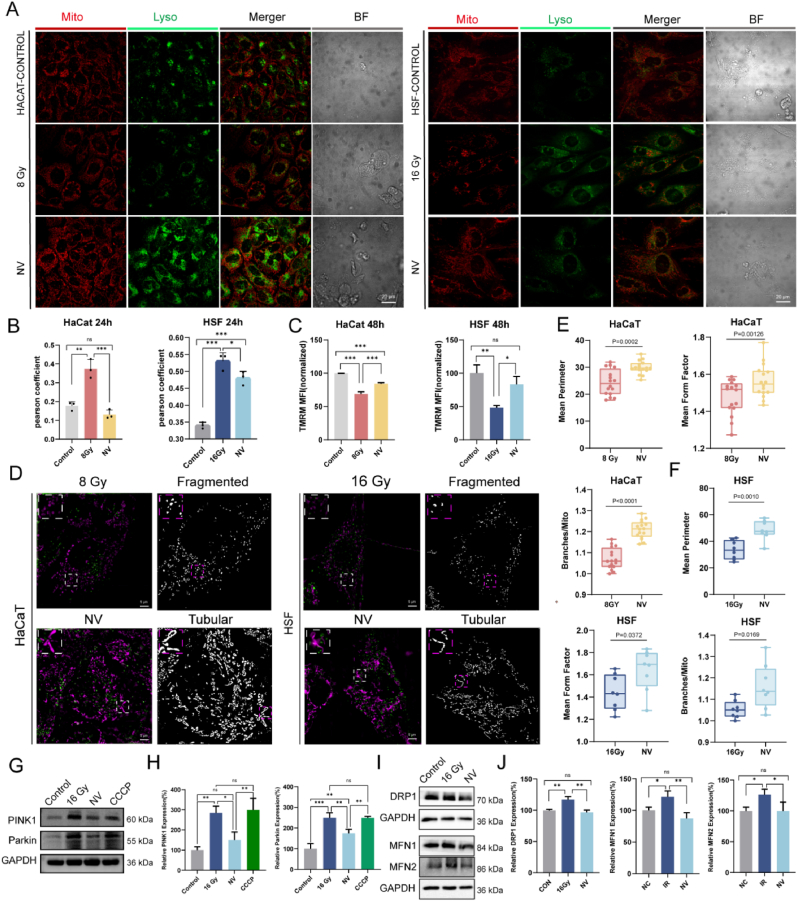
NV inhibit radiation-induced mitophagy and restore mitochondrial dynamics in HaCaT and HSF cells. A) Confocal laser scanning microscopy images showing NV regulates mitochondria(red) co-localization with lysosome(green) in HaCaT(left) and HSF(right). Scale bars: 20 μm. B) The bar graph shows Pearson's coefficient for the co-localization of mitochondria with lysosome of HaCaT (left) and HSF(right), separately(n = 3). C) Mitochondrial membrane potential (Δψm) assessed by TMRE staining at 48 h, with NVs preserving mitochondrial function in both cell types(n = 3). D) Display plots of mitochondria in HaCaT (left) and HSF(right) obtained by super-resolution microscopy and skeleton analysis (fragmented mitochondria after irradiation and rescue of tubular network morphology following NV treatment), white boxed areas are enlarged. Scale bars: 5 μm. E,F) Quantitative analysis of mitochondrial morphology in (E) HaCaT (n = 16 cells per group)and (F) HSF (n = 8 cells per group). The image parameters include the perimeter, which describe the size of the mitochondria, and the form factor and branches/mito. G,H) Western blot and quantification of mitophagy-related proteins PINK1 and Parkin in HSF(n = 3). I,J) Western blot and quantification of mitochondrial dynamics regulators DRP1 (fission), MFN1, and MFN2 (fusion) in HSF. GAPDH was used as a loading control. Data are shown as mean ± SD. ∗P < 0.05, ∗∗P < 0.01, ∗∗∗P < 0.001. (For interpretation of the references to color in this figure legend, the reader is referred to the Web version of this article.)

Confocal imaging (Fig. 6A) revealed increased mitochondrial–lysosomal co-localization in HaCaT (8 Gy) and HSF (16 Gy) cells after X-ray exposure, suggesting enhanced mitophagy. NV treatment reduced this co-localization in both cell types. Quantitative results (Fig. 6B) showed a significant rise in the Pearson's correlation coefficient post-irradiation (HaCaT: 0.36 vs. 0.18, HSF: 0.52 vs. 0.34) and a notable decrease after NV treatment (HaCaT: 0.13, HSF: 0.46). These findings suggest ionizing radiation causes mitochondrial damage and activates mitophagy, with HaCaT cells showing a stronger response. NVs effectively mitigated this process, maintaining mitochondrial integrity. The different responses between cell types may result from varying metabolic needs and repair mechanisms. Excessive mitophagy can lead to a reduction in mitochondrial mass, which may impair the cellular capacity to meet its energy demands, especially during periods of increased metabolic activity such as tissue repair [39,40]. Overall, NVs help regulate mitochondrial quality control. The balance between mitochondrial biogenesis and mitophagy is vital for maintaining energy metabolism homeostasis. Besides, NVs transport process helps maintain mitochondrial integrity and function by delivering mitochondrial components to target cells. This mechanism not only contributes to the delivery of essential proteins required for mitochondrial function, but also contributes to cellular homeostasis and reduces the need for autophagy.

The ΔΨm plays a crucial role in regulating mitochondrial homeostasis, and alterations in ΔΨm significantly affected mitochondrial function [41]. Therefore, TMRE fluorescence was used to monitor ΔΨm measured by flow cytometry (Fig. 6C). NV treatment significantly restores ΔΨm levels in both HaCaT and HSF cells. Previous studies have shown that EVs are capable of being translocated into recipient macrophages and subsequently fusing with the mitochondrial network of target cells [42].Therefore, we speculate that NVs preserve mitochondrial function and bioenergetic capacity following radiation exposure through specific targeting of dysfunctional mitochondria. Mitochondrial activity reduction was associated to the changes mitochondria structure [43]. Therefore, we examined mitochondrial morphology through SIM and used Mitochondrail Analyzer for quantification. Fragmented mitochondrial networks are predominant in both HaCaT and HSF cells (Fig. 6D) characterized by small, swollen mitochondria, indicative of excessive mitochondrial fission. While, NV treatment restores mitochondrial network integrity with decreased fragmented mitochondria, and an increase in long and short tubular mitochondria. Quantitative analysis of mitochondrial morphology in HaCaT cells (Fig. 6E) shows a significant increase in mitochondrial perimeter (p = 0.0002) and form factor (p = 0.00126) following NV treatment, showing significantly enhanced mitochondrial elongation. Similarly, in HSF cells (Fig. 6F), NV treatment leads to an increased mean perimeter (p = 0.0010) and form factor (p = 0.0372) compared to radiation-exposed cells, further supporting the shift towards elongated, tubular mitochondria. Additionally, the branches/mitochondrial mass ratio significantly higher in both skin cells further supporting mitochondrial network recovery, indicating that NVs specifically target mitochondrial morphology rather than altering overall mitochondrial content. The mechanisms of MQC are complex, in addition to mitophagy, in which mitochondrial fission/fusion kinetics are very important [44]. Radiation can induce mitophagy by increasing ROS in mitochondria. PINK1, as a mitochondrial kinase, accumulates on the outer mitochondrial membrane when the mitochondrial membrane potential decreases, and recruits Parkin (an E3 ubiquitin ligase) to the mitochondrial surface. The recruitment of Parkin further promotes the ubiquitination of damaged mitochondria, thereby marking these mitochondria for autophagic degradation [45,46]. Maintaining mitochondrial homeostasis relies on the functional connection between mitophagy and mitochondrial dynamics [47]. Western blot analysis revealed a significant upregulation of PINK1 and Parkin proteins following irradiation (Fig. 6G and H), consistent with activation of the PINK1–Parkin pathway and initiation of mitophagy in response to mitochondrial damage. On the contrary, NV treatment markedly suppressed the expression of both proteins, displaying an inhibitory effect on radiation-induced mitophagic signaling. We used CCCP as a canonical depolarizing agent, which is known to stabilize PINK1 on the OMM and recruit/activate Parkin to initiate mitophagy, as established by multiple high-impact studies [48,49]. The protein expression of both PINK1 and Parkin shows no difference in 16 Gy group and the CCCP group. Based on the Western blot analysis, Pearson correlation analysis was conducted to evaluate the correlation of protein expression levels between the 16 Gy group and the CCCP group (Fig. S4A and B). The statistical analysis show that the expression levels of these two groups exhibited strong correlation in both Pink1 (r = 0.9753, R^2^ = 0.9513, n = 3) and Parkin(r = 0.9854, R^2^ = 0.9711, n = 3). This observation suggests that the expression patterns of PINK1 and Parkin in the CCCP and 16 Gy groups are similar. These results suggest that NVs contribute to the preservation of mitochondrial integrity by limiting aberrant autophagy activation under radiation stress. Consequently, NV-mediated modulation of the PINK1–Parkin axis may serve as a protective mechanism to maintain mitochondrial homeostasis and mitigate cellular injury following irradiation. Moreover, Western blot analysis (Fig. 6I) also evaluates the expression of fusion and fission proteins. DRP1 induces cleavage of the outer membrane of mitochondria, splitting them into small fragments that may help to remove damaged mitochondria. However, excessive or uncontrolled mitochondrial division may give rise to mitochondrial overabundance, aberrant morphology and dysfunction. High dose X-ray (16 Gy) significantly upregulates DRP1 expression in HSF, promoting mitochondrial fragmentation and dysfunction (p < 0.01). NV treatment reduces DRP1 expression, suggesting that NVs suppress excessive mitochondrial fission and contribute to mitochondrial homeostasis. GAPDH is used as a loading control, confirming equal protein loading. The MFN1 and MFN2 are substrates for Parkin [50]. The failure of mitochondria to maintain regulated fusion following cellular injury results in aberrant alterations in mitochondrial morphology, such as excessive fusion leading to the formation of ‘giant mitochondria’ [45]. This observation implies that such excessive fusion may disrupt mitochondrial function, subsequently prompting the removal of surplus mitochondria via the autophagy pathway. Interestingly, heightened mitochondrial fusion may activate autophagic processes to eliminate mitochondria, culminating in a condition characterized by ‘excessive autophagy’.

These in vitro and in vivo studies demonstrates that ME-NVs re-establish MQC under radiation stress. NVs act through two complementary mechanisms. First, they suppress PINK1/Parkin overactivation and the associated aberrant mitophagy, preserving functional mitochondria. Second, they correct DRP1/MFN/OPA1-mediated fission–fusion imbalance, thereby restoring ΔΨm and network organization.

### 3.7 DIA-based proteomic analysis of NVs promoting DNA repair and regulating mitochondrial functions

**Fig. 7:**
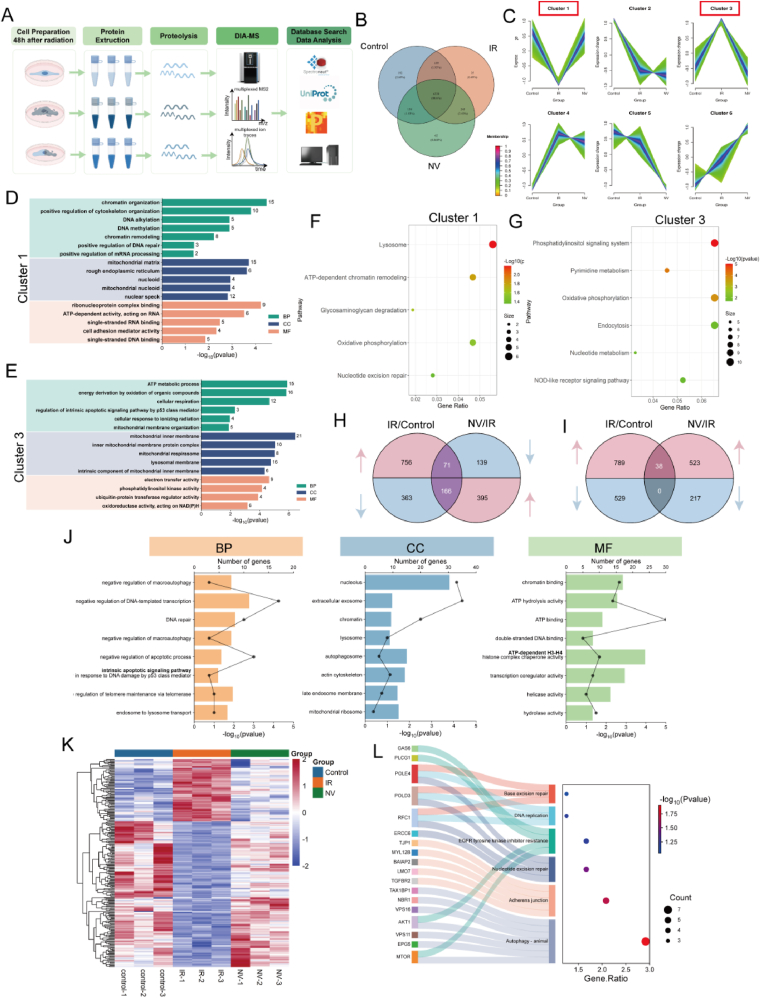
DIA-based proteomic analysis of NVs promoting DNA repair and regulating mitochondrial functions. A) Schematic workflow of DIA-MS-based quantitative proteomics for HSF(Created by Figdraw, ID:YYYWT9aeae). B) Venn analysis among control, irradiated (IR), and NV-treated groups(NV). C) fuzzy clustering analysis revealed distinct expression dynamics across six clusters. D-G) GO enrichment and KEGG analysis of Clusters 1 and 3. H,I) Venn diagrams of pairwise group comparisons illustrate unique and shared DEPs among IR/Control and NV/IR groups. J, K) GO enrichment, hierarchical clustering of shared 237 DEPs. L) Sankey-Bubble diagram depicting highly enriched KEGG pathways and the hub genes within 237 DEPs.

To further elucidate the potential mechanisms of ME-NVs in radiation induced skin injury, we conducted an Astral mass spectrometer-based DIA (Data-Independent Acquisition) technology for proteomics research on three HSF cell groups: NC, IR, and NVs (Fig. 7A). This method combines Astral's sensitivity with DIA's high throughput, achieving a wide dynamic range in protein quantification (Fig. S5A). High confidence scores near 1.0 indicate reliable peptide identification (Fig. S5B), and peptide sequence lengths means efficient digestion (Fig. S5C). Overall, the ASTRAL-DIA workflow effectively identifies and quantifies low-abundance proteins in complex samples.

A Venn diagram (Fig. 7B) shows the distribution of proteins across three groups: 35 proteins (0.49 %) were unique to the IR group termed IR-specific proteins, while 138 proteins (1.93 %), termed NV-rescued proteins, were common to the Control and NVs groups but absent in the IR group (Fig. S5D). GO enrichment analysis showed that IR-specific proteins are mainly enriched in extracellular matrix degradation, inflammatory signaling, and apoptosis, aligning with cellular remodeling and cell death under radiation stress (Fig. S5E). These proteins are localized to the cytoskeleton and membranes, focusing on actin organization and protease activity. Conversely, NV-rescued proteins are enriched in DNA repair, mitochondrial function, and metabolic adaptation, accumulating in the nucleus, mitochondria, and chromatin, indicating genome maintenance and energy metabolism recovery. Their functions include nucleic acid binding, oxidoreductase activity, and ATPase functions, essential for post-injury recovery.

To elucidate the pathways through which NVs facilitate functional recovery, we performed enrichment analysis using the KEGG and WikiPathways databases. The resulting bubble chart (Fig. S5F) reveals significant enrichment of NV-regulated proteins in key DNA damage response pathways, including "DNA IR damage and cellular response via ATR" and "DNA repair pathways full network." These results suggest that NVs contribute to the preservation of genomic integrity by enhancing DNA repair mechanisms, potentially mitigating radiation-induced genomic instability.To systematically characterize protein expression dynamics in response to radiation injury and NV treatment, we applied the Mfuzz soft clustering algorithm to analyze 6330 proteins across the three experimental groups, identifying six clusters with distinct temporal expression patterns (Fig. 7C). Each cluster exhibited specific regulatory trends associated with injury progression and therapeutic intervention. Cluster 1 proteins, initially suppressed by injury but restored with NV treatment, were linked to DNA repair, protein folding, and apoptosis inhibition (Fig. 7D). KEGG analysis indicated their involvement in lysosome activity, protein processing, oxidative phosphorylation, and metabolic pathways (Fig. 7F), underscoring their role in cellular homeostasis and energy metabolism post-NVs intervention. Conversely, Cluster 3 proteins were upregulated after injury and downregulated by NV treatment, associated with oxidative stress, mitochondrial dysfunction, and injury response (Fig. 7E). KEGG pathway enrichment highlighted their roles in apoptosis, oxidative stress response, and injury-related signaling (Fig. 7G). These findings offer insights into injury response and NV treatment effects, identifying potential targets for further research. Volcano plots (Fig. S5G and H) showed that radiation caused extensive proteomic changes, which NV treatment partially reversed, especially in proteins related to mitochondrial function and stress responses. Moreover, GSEA analysis was consistent with above results, revealing that mitophagy was inhibited (Fig. S5I), while DNA repair capacity (Fig. S5J) was promoted in NVs group compared with IR group.

237 proteins were differentially expressed in both comparisions. Of these, 71 proteins were upregulated by radiation but suppressed by NVs, while 166 proteins were downregulated by radiation but restored with NV treatment, termed Rev-RSI proteins (Fig. 7H). Only 38 proteins were consistently upregulated, and none were consistently downregulated, named Pro-RSI proteins (Fig. 7I). GO analysis revealed that Rev-RSI proteins were mainly involved in DNA repair, apoptosis regulation, and autophagy suppression, notably in p53-mediated apoptotic signaling and macroautophagy inhibition (Fig. 7J). Hierarchical clustering analysis demonstrated that proteins down-regulated by IR exposure were often restored or upregulated after NV treatment, while some proteins upregulated in the IR group normalized or decreased in the NVs group (Fig. 7K). 237 Rev-RSI proteins were enriched in DNA damage repair mechanisms, including base excision repair, nucleotide excision repair, and DNA replication. The enrichment of DNA repair proteins such as POLD3, POLE4, RFC1, and ERCC6 in our dataset aligns with established literature indicadting that mitochondrial ROS amplification exacerbates genomic instability, whereas restoration of mitochondrial homeostasis indirectly promotes DNA repair [51,52]. Of particularly note, the autophagy pathway was significantly enriched, with critical regulators such as *AKT1, MTOR, EPG5, VPS11, VPS16, NBR1* and *TAX1BP1* implicated in this process (Fig. 7L). These proteins may contribute to mitochondrial quality control and lysosome-mediated degradation. In summary, these findings emphasize the relevance of DNA repair, autophagy regulation, and intercellular signaling in the proteomic response to radiation injury and its modulation by NV treatment.

Radiation can initiate a series of intricate cellular stress responses, characterized by mitochondrial dysfunction, impaired DNA damage and repair mechanisms, and a marked increase in apoptosis [[53], [54], [55]]. It induces substantial ROS production, which directly damages the mitochondrial membrane. Our observations revealed a significant upregulation of autophagy receptors NBR1 and TAX1BP1 [56], alongside increased levels of fusion regulators such as EPG5*,* VPS11, and VPS16 [[57], [58], [59]]. These findings suggest an abnormal enhancement of autophagic flux, coupled with an imbalance in mitochondrial function, ultimately resulting in mitochondrial depletion and metabolic disorders. Concurrently, there was a notable upregulation of AKT1 and mTOR expression, indicating aberrant activation of the PI3K/AKT/mTOR pathway [[60], [61], [62]]. While this pathway may facilitate short-term cell survival, prolonged activation of mTOR inhibits ULK1 activity, thereby obstructing the initiation of autophagy [63,64]. This inhibition leads to the accumulation of damaged organelles and proteins, exacerbating mitochondrial stress and dysfunction, and further promoting apoptosis.

NVs effectively inhibited the aberrant activation of AKT1 and mTOR, potentially mitigating cellular and mitophagy impairment in HSFs and thereby reducing the accumulation of damaged organelles and proteins. NVs are abundant in mitochondrial proteins, including mitochondrial metabolism enzymes such as ATP5F1A/B, COX7A2L, and NDUFS8 [[65], [66], [67]], which enhance ATP synthesis, stabilize membrane potential and support mitochondrial energy metabolism at the source. NVs also contain antioxidant proteins such as SOD2, GPX4, and members of the PRDX family [68], efficiently scavenge excess ROS and inhibit ROS-mediated PINK1 activation. The activation of PINK1 reduced radiation-induced mitochondrial stress and abnormal autophagy initiation. Furthermore, the down-regulation of key autophagy-related factors, including NBR1, TAX1BP1, EPG5, VPS11 and VPS16 may help restore the dynamic balance of autophagy regulation, alleviate mitochondrial dysfunction, and enhance cellular metabolism following NV treatment.

Beyond the mechanisms directly demonstrated in our study, accumulating evidence has linked mitophagy, mitochondrial quality control, mitochondrial–ER stress, and the cell fate decisions through well-defined molecular axes. These insights may further elucidate the therapeutic potential of ME-NVs. Recent studies have identified PHB2 as a central regulator coordinating mitophagy and mitochondrial dynamics. One nanomedicine delivery system modulates MQC via the PIEZO1-TMBIM6-PHB2 axis, reducing inflammation and suppressing pyroptosis [69]. Similarly, certain bioactive compounds act at mitochondria-endoplasmic reticulum contact sites (MERCs) through the DUSP1-PHB2 axis, coordinating MQC with ER-phagy to restore cellular homeostasis [70]. Additionally, some small molecules regulate the mitochondrial–ER unfolded protein response via the Sirt5-Mettl3 axis, promoting mitochondrial biogenesis by restoring PGC-1α/TFAM/Nrf-1 signaling [71]. Apart from the canonical ubiquitin-dependent PINK1-Parkin pathway, mitophagy can also occur through receptor-mediated routes such as those involving FUNDC1. Moderate mitochondrial depolarization results in excessive fission and impaired mitophagy, mediated by NR4A1-Mff and inhibition of FUNDC1. In contrast, pronounced depolarization or extensive protein ubiquitination preferentially activates PINK1-Parkin-dependent mitophagy [72]. Furthermore, FUNDC1-mediated mitophagy couples with the mitochondrial unfolded protein response (UPRmt), enabling an integrated MQC response [69,73].

Together, integration of our proteomic data with recent literature suggests that mitochondria-enriched NVs not only act as ROS scavengers but also serve as broad regulators of mitochondrial quality control, ER-mitochondria crosstalk, DNA repair, and multi-modal cell death suppression, thereby providing a comprehensive protective mechanism against radiation-induced skin injury and other oxidative stress-related disorders.

Our findings highlight the therapeutic potential of mitochondria-enriched nanovesicles in mitigating RSI. These insights underscore that targeted regulation of mitochondrial homeostasis may represent a viable and innovative strategy for clinical intervention. While further studies are required to validate key pathways (e.g., PINK1-Parkin, PHB2) and to confirm PINK1/Parkin mitochondrial accumulation by fractionation or targeted enrichment, as well as to clarify the role of NV-derived mitochondrial components.

## 4 Conclusion

**Fig. 8:**
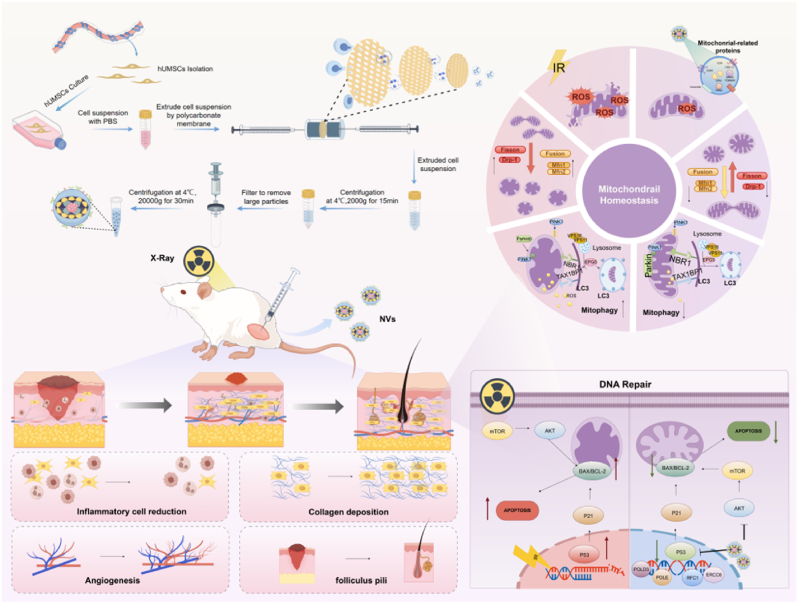
Schematic illustration of the fabrication of Mitochondria-Enriched Nanovesicles derived from hUMSCs(ME-NVs) and their application in treatment of radiaiton induced skin injury((Created by Figdraw, ID:SWSSP5444b).

In this study, we developed mitochondria-enriched nanovesicles (ME-NVs) exhibiting high biocompatibility and targeted delivery capacity. Using both cell and animal models exposed to different radiation doses, we demonstrated morphological and functional mitochondrial changes associated with the early progression of ARD. The multifunctional NVs effectively treated radiation-induced skin injury by reducing inflammation, promoting collagen deposition, rescuing angiogenesis, and regenerating follicles in vivo, while alleviating mitochondrial dysfunction, enhancing DNA repair, and inhibiting apoptosis. In vitro, NVs protected against radiation-induced skin injury by reducing apoptosis, promoting DNA repair, and regulating PINK1/Parkin-mediated mitophagy. NVs also restored mitochondrial homeostasis by balancing mitochondrial fission and fusion dynamics. DIA-based proteomic analysis support the therapeutic potential of NVs in mitigating radiation-induced cellular damage through comprehensive regulation of mitochondrial integrity and function(Fig. 8).

## CRediT authorship contribution statement

**Mengru Zhu:** Writing – original draft, Funding acquisition, Formal analysis, Data curation. **Junhao Xia:** Methodology, Formal analysis, Data curation. **Jia Liu:** Methodology, Data curation. **Wei Zou:** Writing – review & editing, Supervision, Investigation. **Xin Guan:** Visualization, Validation, Investigation. **Lizhi Wang:** Visualization, Validation, Investigation. **Yichen Wang:** Writing – original draft, Data curation. **Bing Wang:** Methodology, Data curation. **Fengya Wang:** Methodology, Funding acquisition. **Qingwen Zhang:** Software, Methodology. **Keman He:** Software, Methodology. **Lukuan Liu:** Writing – review & editing, Supervision, Resources, Investigation, Conceptualization. **Jing Liu:** Writing – review & editing, Supervision, Resources, Methodology, Funding acquisition, Conceptualization.

## Declaration of competing interest

The authors declare the following financial interests/personal relationships which may be considered as potential competing interests: Lukuan Liu reports financial support was provided by 10.13039/501100001809National Natural Science Foundation of China. Lukuan Liu reports financial support was provided by the First Affiliated Hospital of Dalian Medical University and 10.13039/501100002979Dalian Institute of Chemical Physics (DMU-1 & 10.13039/501100002979DICP Joint Program). Jing Liu reports financial support was provided by 10.13039/501100008990Liaoning Directed Project for Planning of Science and Technology. Jing Liu reports financial support was provided by Dalian High-level Talent Team Project. Mengru Zhu reports financial support was provided by Dalian Science and Technology Talent Innovation Support Program. Fengya Wang reports financial support was provided by 10.13039/501100005047Natural Science Foundation of Liaoning Province. If there are other authors, they declare that they have no known competing financial interests or personal relationships that could have appeared to influence the work reported in this paper.

## Data Availability

No data was used for the research described in the article.
