# Supplementary material for: Mitochondria-enriched nanovesicles: A novel approach for treating radiation-induced skin injury
Source: Mater Today Bio. 2025 Oct 8;35:102377. doi: 10.1016/j.mtbio.2025.102377 (PMC12552567; doi:10.1016/j.mtbio.2025.102377)
Supplement: Multimedia component 1 [file mmc1.docx]

Supporting Information

Comparison of Protein Yield Between NV and sEV

Table S1. Protein yield was quantified from equal numbers of cells. Data are presented as mean ±SD, n=3. Statistical analysis was performed using an unpaired two-tailed t-test.

| Group | Protein yield(μg/10^7^ cells) | P value |
| --- | --- | --- |
| NV | 141.2± 4.5 | <0.0001 |
| EV | 9.3± 1.0 |  |


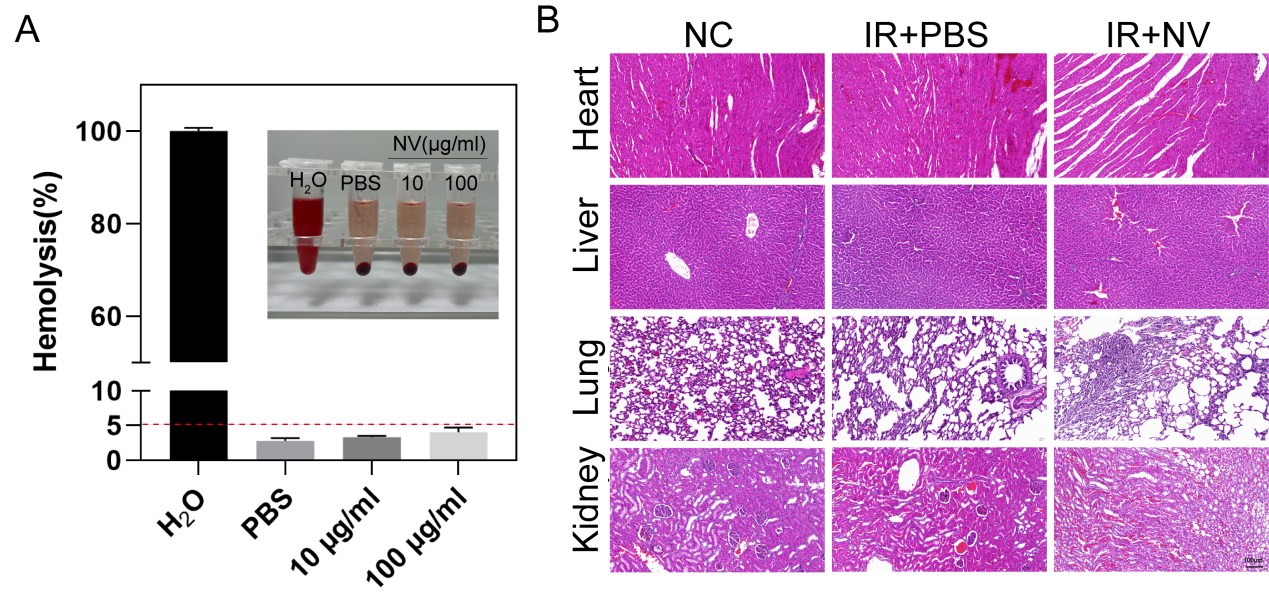


Figure S1. A) Hemolysis rates of low and high concentration of NV. PBS: negative control group. distilled water(H_2_O): positive control group. Inset: Digital photo showing hemolysis phenomenon. B) H&E-stained images of the heart, liver, spleen, lung and kidney of each group on day 28 after radiation. Scale bars:100 μm.


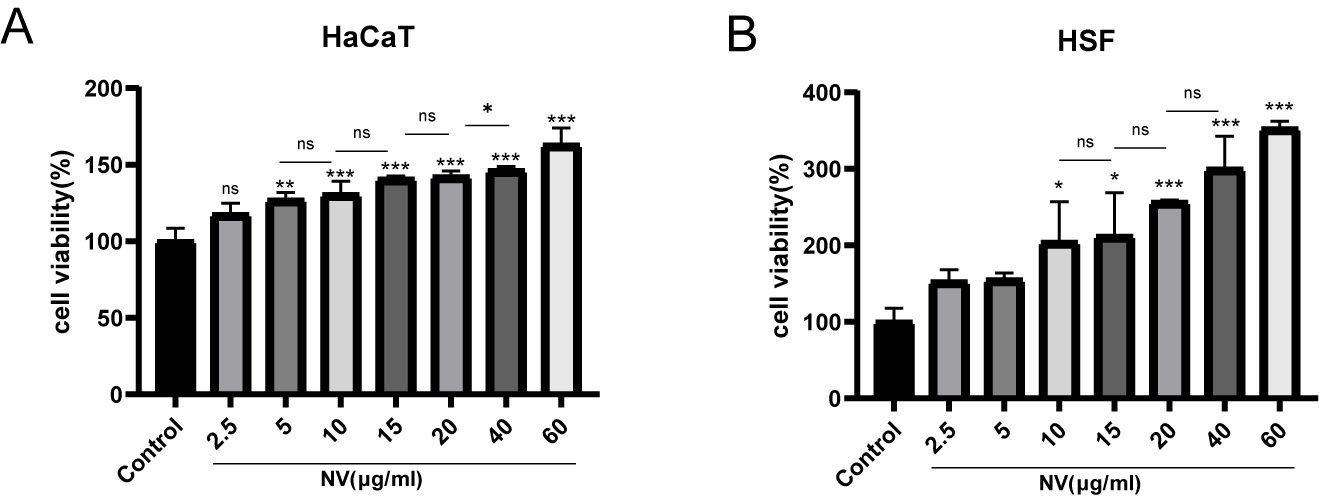


Figure S2. NVs exhibit no cytotoxicity and promote cell viability. Cell viability of A)HaCaT and B) HSF was tested at different concentrations of NVs (2.5–60 μg/ml) after an incubation period of 24 h by CCK-8 assay. Data are presented as mean ± SD (n = 6). *P < 0.05, **P < 0.01, ***P < 0.001 vs. Control, ns: p > 0.05.


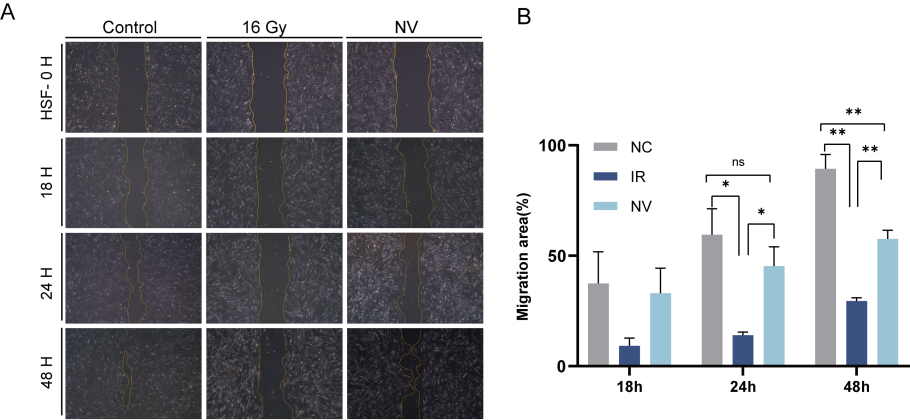


Figure S3. A) Representative images of migration assay and the image was taken at the indicated times (scale bar,100 μm). B) Quantitation of migration assays of HSF. n = 3, ns, not significant, p > 0.05, *P <0.05, **P <0.01.


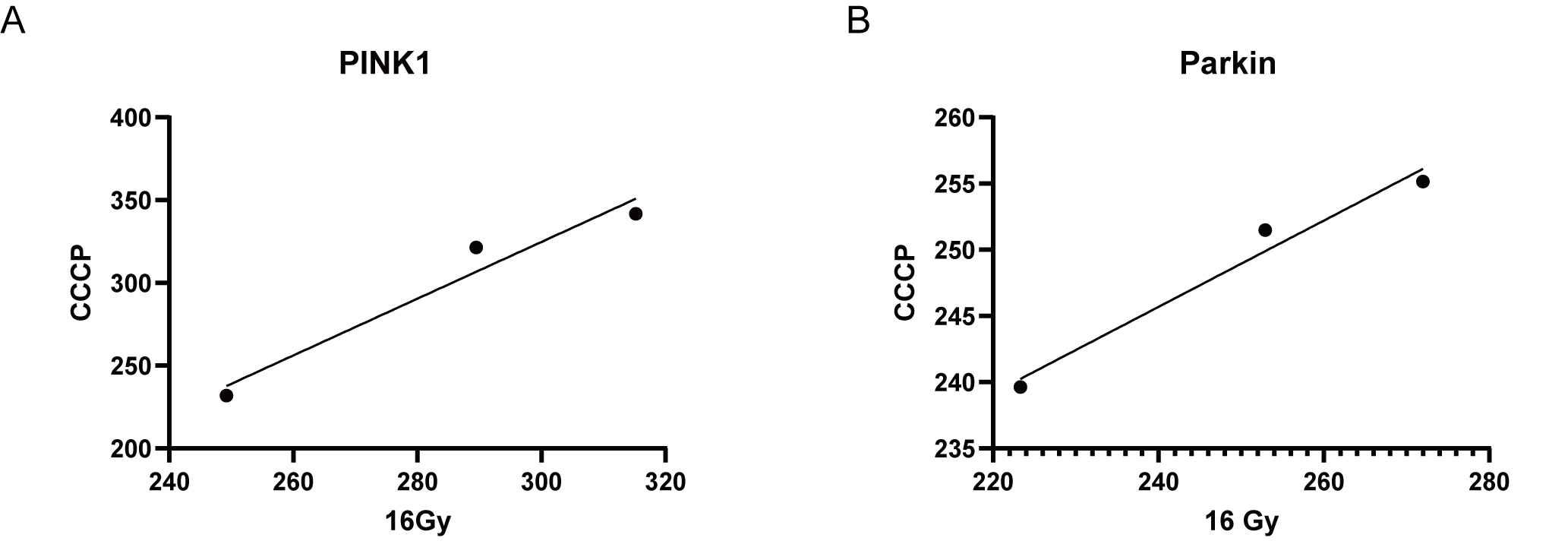


Figure S4. Pearson correlation analysis show that the expression levels of 16 Gy group and CCCP group exhibited strong correlation in both A) PINK1 (r =0.9753, R²= 0.9513, n=3) and B) Parkin(r = 0.9854, R²=0.9711, n=3). r = Pearson correlation coefficient.


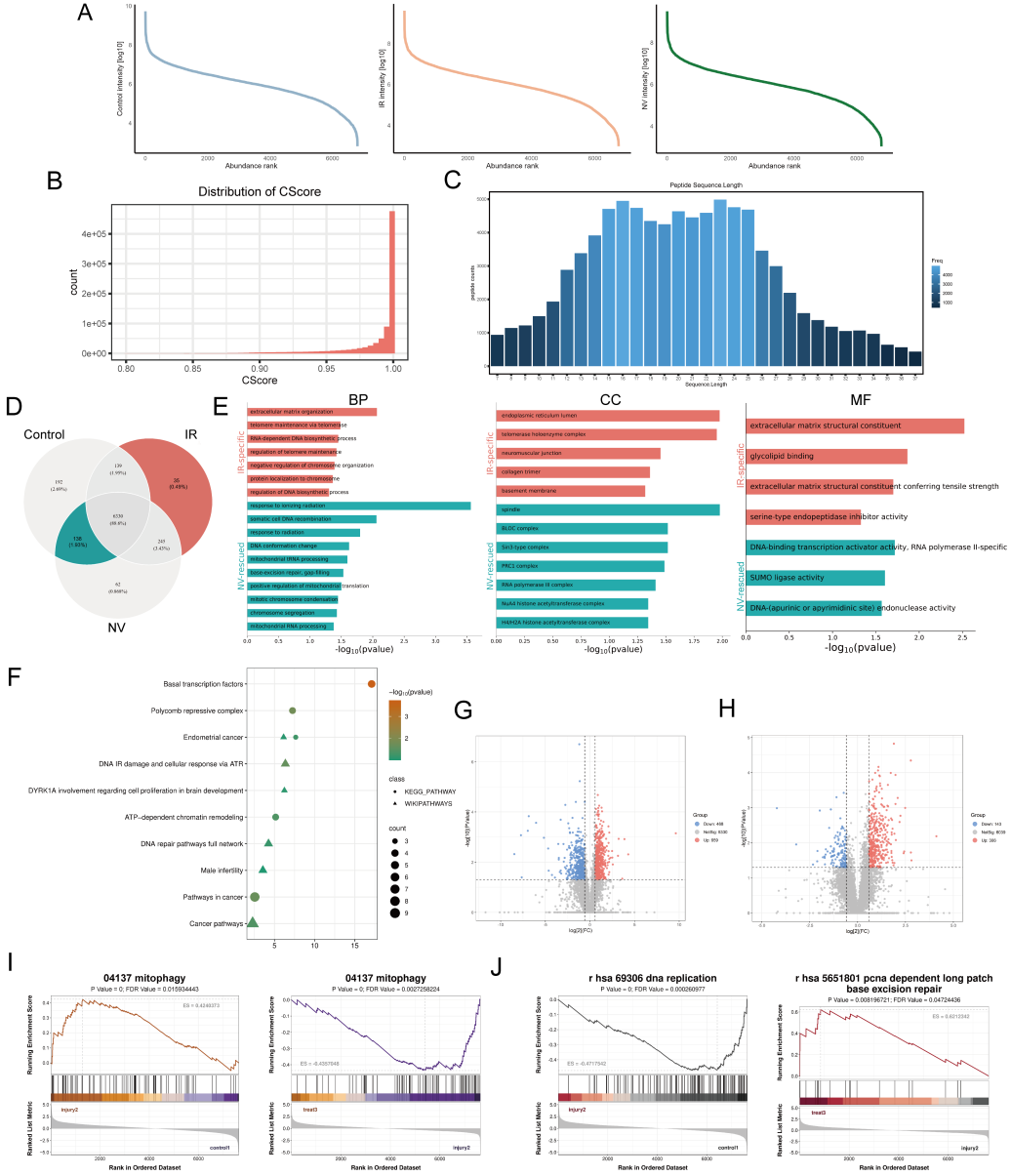


Figure S5. Proteomics analysis of HSF radiation damage and treatment. A) Sequencing depth analysis based on DIA-MS data. B) Distribution of CScore across all peptides identified by Spectronaut. C) Histogram of peptide sequence lengths colored by frequency. D) Venn analysis highlight 35 IR-specific proteins (red) in IR group and 138 NV-rescued proteins(green) shared among Control and NV groups. E,F) GO enrichment and Functional enrichment dot plots using KEGG and WikiPathways databases. G,H) Volcano plots comparing differential expression of IR/Control and NV/IR groups. I,J) GSEA of mitophagy pathway and DNA repair related pathways in IR/Control and NV/IR groups.
